# Whole systems approach to diet and healthy weight: a longitudinal process evaluation in East Scotland

**DOI:** 10.1177/17579139231203858

**Published:** 2023-10-30

**Authors:** G Breslin, W Wills, C Bontoft, O Fakoya, H-A Greco, N Lloyd, AP Wagner, A Wellings, S Harding, KE Brown

**Affiliations:** School of Psychology, Queen’s University Belfast, Belfast, UK; School of Health and Social Work, University of Hertfordshire, Hatfield, UK; Applied Research Collaboration (ARC) East of England, National Institute for Health and Care Research (NIHR), London, UK; Department of Psychology, Sport and Geography, School of Life and Medical Sciences, University of Hertfordshire, College Lane Campus, College Lane, Hatfield AL10 9AB, UK; Department of Psychology, Sport and Geography, School of Life and Medical Sciences, University of Hertfordshire, Hatfield, UK; Department of Psychology, Sport and Geography, School of Life and Medical Sciences, University of Hertfordshire, Hatfield, UK; Department of Psychology, Sport and Geography, School of Life and Medical Sciences, University of Hertfordshire, Hatfield, UK; Applied Research Collaboration (ARC) East of England, National Institute for Health and Care Research (NIHR), London, UK; Norwich Medical School, University of East Anglia, Norwich, UK; Department of Psychology, Sport and Geography, School of Life and Medical Sciences, University of Hertfordshire, Hatfield, UK; Department of Psychology, Sport and Geography, School of Life and Medical Sciences, University of Hertfordshire, Hatfield, UK; Department of Psychology, Sport and Geography, School of Life and Medical Sciences, University of Hertfordshire, Hatfield, UK

**Keywords:** systems approach, obesity, policy, nutrition, health

## Abstract

**Aims:**

Obesity contributes to morbidity and early mortality, affecting people of all ages and sociodemographic backgrounds. Despite attempts to address obesity, efforts to date have only had limited success. Adopting a whole systems approach (WSA) may potentially address obesity and emphasise complex inter-relating factors beyond individual choice. This study aimed to assess implementation of WSA to diet and healthy weight in two council areas of Scotland, longitudinally exploring enablers and barriers. One area followed a Leeds Beckett WSA model (LBM) of implementation, while the other used a hybrid model incorporating existing working systems.

**Methods:**

To assess the process of implementing a WSA, interviews and focus groups were conducted after initiation and 1 year later.

**Results:**

Main enablers included: belief in WSA effectiveness; positive relationships between key personnel; buy-in at community and national levels; funding availability; the working group responsible for coordinating the system development comprising individuals with diverse expertise; good communication; and existing governance structures. Barriers included: insufficient funding; high staff turnover; inadequate training in WSA methodology; engaging all relevant stakeholders and reverting to ‘old ways’ of non-WSA working. The LBM provided a framework for system setup and generating an action plan.

**Conclusion:**

This study provides the first independent longitudinal process evaluation of WSAs that have incorporated Leeds Beckett methodology, and offers insights into how a WSA can be implemented to address diet and healthy weight.

## Introduction

The world faces an obesity epidemic that contributes considerably to morbidity and early mortality.^1^ Obesity affects people of all ages and backgrounds, but it can exacerbate health disparities because members of sociodemographic and ethnic groups that experience poorer health outcomes are more likely to be impacted by obesity.^2^ In Scotland in 2019, 66% of adults were affected by overweight, and of these 29% were affected by obesity. Prevalence of overweight including obesity was significantly higher among men compared with women (69% and 63%, respectively). Overweight and obesity rates were 40% of those aged 16–24, and 79% of those aged 65–74. Obesity rates are higher in the most deprived areas, particularly for women; 40% of women in the most deprived areas of Scotland experience obesity compared to 18% in the least deprived.^3^ Despite attempts to address this epidemic through policy and intervention, success has been limited.^4^ Limited impact may be explained in part because single policies (e.g. introduction of a levy on sugary drinks) and intervention targeted at specific population segments (e.g. family weight management services) fail to address the complex array of interacting factors identified as causally impacting on population obesity.^5^ Adopting a whole systems approach (WSA) to address influences on diet and healthy weight has been identified as having potential to tackle this complex area.^6,7^

A WSA incorporates a range of comprehensive initiatives targeted at system change by reaching government, policy decision makers, individuals, groups and community-level environments and drivers of human action.^8^ A recent systematic review of 65 studies examining implementation and effectiveness of WSAs, 33 of which focused on obesity, identified improved outcomes including: body mass index (BMI) reductions; increased parental and community awareness; community capacity building; nutrition and physical activity environment changes; and improved safety and wellbeing of community members.^9^ Our recent review-of-reviews of WSAs applied to diet, healthy weight and obesity^4,10^ showed, however, that evidence for WSA effectiveness remains in its infancy, but some case studies where WSA were effective may aid new WSA adopters.^9^ This echoes findings in an evidence synthesis of a WSA to obesity prevention, serving to inform the Northern Ireland Obesity Prevention Strategy^11^ and the recent Academy of Medical Science report on what is next for WSAs to public health.^12^ Therefore, there remains a need for robust longitudinal evidence to strengthen WSAs in government policy and practice.^4,10,11^

In addition, there is a paucity of evidence on factors important for successful WSA set-up and implementation. The National Institute for Health Care and Excellence (NICE) commissioned an evidence review of WSAs to obesity to inform the delivery of WSAs.^13^ They noted that authentic WSAs draw on complexity science to explain how system features interact. The authors proposed 10 features of a WSA. However, from among 13 WSA studies that focused on obesity, Bagnall et al.^9^ identified that success did not necessarily require all 10 features,^9,10^ a conclusion supported by others.^11^

To empower public health leaders to utilise a WSA to tackle diet and unhealthy weight, Public Health England^6^ developed a guiding framework for WSA set-up, often labelled the ‘Leeds Beckett Model’ (LBM). In 2019, Public Health Scotland launched a WSA pilot project in Scotland and provided funding to support local authority regions to set-up WSAs. Process evaluation of the pilots demonstrated how local systems can work more effectively to address complex public heath challenges.^14^ In addition, WSA training has accelerated WSA interest. Areas adopting WSAs made progress in establishing new ways of working despite COVID-19 challenges.^14^

In this study, conducted in Scotland, two local authority (LA) areas receiving WSA funding, but not included in the national evaluation, were selected. This provided an opportunity to evaluate WSA implementation, including comparison of different implementation models (i.e. the untested LBM versus a hybrid model). This study assessed implementation of a WSA to diet and healthy weight and longitudinally explored enablers and barriers. Collected data also informed the range and extent of activity conducted by stakeholders in WSA delivery.

## Methods

### Design

Focus groups and interviews were conducted with members of relevant WSA Core Working Groups (CWGs) and wider stakeholder networks in two LAs in East Scotland. CWGs are responsible for coordinating the local WSA with stakeholders, who were invited to participate in LA-led workshops to inform WSA implementation. Two council areas (labelled A and B to maintain confidentiality) were selected from among five potential localities. Selection was driven by their contrasting choice of methodology in implementing a WSA: Location A developed a hybrid WSA model without following specific guidance – details are presented in the next section, while Location B utilised the LBM.^6^ Location descriptions and population demographics are included in the study protocol, alongside further study detail.^4^

Focus groups and interviews sought to explore: how stakeholders got involved with the WSA and their initial understandings of them; the process and experience of WSA implementation; enablers of and barriers to WSAs. Focus groups and interviews had been planned for three time points, with the third intended to collect data about implementation of WSA action plans. However, COVID-19-related implementation delays necessitated data collection ceasing after two time points. Time point 1 interviews and focus groups were conducted after each area’s CWG establishment, and after their first workshop at which participants develop a local ‘map’ of the causes of dietary behaviour and healthy weight in their area. Time point 2 interviews were conducted after the second LA workshop and development of the WSA action plan in each area. Due to COVID-19, there was approximately 12 months between these workshops. Consequently, stakeholder interaction and initial momentum for action plan development was reduced.

CWG members participating in focus groups and/or interviews, along with stakeholders attending first workshops in each locality, were also invited to complete monthly surveys consisting of questions relating to: Ecological Momentary Assessment (EMA);^15^ recording WSA delivery activities; and time spent on them. Response rates were low (Supplementary Table S1) and consequently we do not consider the EMA data further; however, as EMA has not been used in this setting previously, the Supplementary materials section include details about the utilised EMA to inform other researchers considering these techniques.

### Model of WSA implementation

In Location A, a WSA was implemented based on existing partnership working practice alongside development of a new obesity map and action plan targeting diet and healthy weight. Two workshops were held to develop the obesity map and subsequently the action plan with stakeholders. While training was received by some members of the CWG in WSA development using LBM, the LBM was not included to guiding activities in this location. We have referred to Location A’s approach as a hybrid model because they structured workshops on the basis of achieving an obesity map through discussions, and developed the action plan through stakeholder involvement of existing partnerships, they did not follow a pre-defined framework or model unlike Location B.

**Figure 1:**
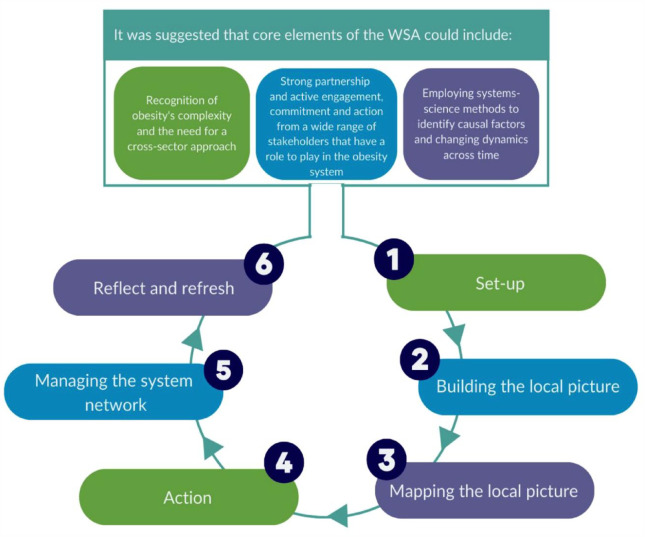
Core elements of the Leeds Beckett Methodology (reproduced with permission)^11^

In Location B, the LBM was operationalised and implemented^6^ – it has six distinct non-linear phases and consists of core elements that are required to support the phases (see Figure 1).

To facilitate LBM delivery, training was provided on its theory and practical phases of model implementation. Our evaluation research team were not involved in this training. Enablers in Location B engaged in training and followed the protocols described in the implementation manual.^6^ Those WSA CWG members joining post initial training were provided with the implementation manual. At completion of our data collection, phases 1–4 had been implemented (see Figure 1).

### Participants and data collection

Participants were recruited from the WSA CWGs and a list of workshop attendees, 32 email addresses were provided and therefore contacted. These potential participants were emailed an invitation, which included a link to further information and a Participant Information Sheet and Consent Form, hosted via REDCap,^16^ a secure online research study management platform. Nineteen participants went on to take part in this evaluation (focus groups: *n* = 14; Location A *n* = 5, Location B *n* = 8, both = 1. Interviews: *n* = 2; Location A *n* = 1, Location B *n* = 1. Monthly surveys only *n* = 3; Location A *n* = 1, Location B *n* = 2). Of the 14 participants who took part in a focus group or interview, 11 also completed the monthly surveys (Location A = 7, Location B = 4). Three people took part in the monthly surveys only (Location B *n* = 2, both = 1).

Thirteen members of the CWG took part in either a focus group or interview (Location A *n* = 6, Location B *n* = 7). The participants included those working in public health management roles, project coordination, public health practitioners, community planning, community regeneration, community development, active schools’ coordinators, post-primary school teachers, and social workers. All focus groups and interviews were conducted via Microsoft Teams, audio-recorded, and transcribed verbatim.

### Data analysis

Focus group and interview transcripts were analysed using framework analysis,^17^ a structured, systematic approach to summarising and analysing qualitative data. Creating the codebook from which to code subsequent transcripts used both inductive and deductive methods, accommodated by framework analysis.^18^ First, the team members familiarised themselves with the data and conducted open coding of initial transcripts. They then met to discuss their codes and incorporate them into a framework.

Included in this team were three Public Involvement in Research group (PIRg) members who had particular interest in assisting this evaluation. After undergoing training in framework analysis, they were integrated into all stages of analysis. The PIRg is composed of 11 members of the public who act as lay people to support the research process and offer expertise through lived experience.

To further refine the framework deductively, data from a systematic review of the literature on implementation and evaluation of WSAs to diet and healthy weight was incorporated,^4^ and information provided in Public Health England’s^19^ guidance on WSAs was also used. This information provided the research team with details on the core components of the LBM and examples of components of previously implemented WSAs. The process of developing the framework was iterative, with further analysis being used to refine the framework. Interpretation of data took a deductive approach, applying themes to each of our research questions.

To provide a broad understanding of the magnitude and range of contribution of staff time to WSA set-up, we totalled activity timings reported on the monthly surveys. We present total timings by activity and overall, separately for each area and exclude individuals working across areas. Some activities categorised as ‘other’ were regrouped. To provide ‘an order of magnitude’ indicative costs of employing staff to deliver these activities, we multiply total activity time by the hourly rate of employing a Social Worker in adult services of £42/h.^20^ This is for the cost year 2021/2022, and includes salary oncosts and overheads, above the base annual salary of £36,000 – see the study by Jones et al.^20^ for further details. Given the wide range of professions (administrator to teacher) and seniority (graduate to trust manger) of staff surveyed precluded, we do not conduct a more precise costing.

## Results

### Qualitative findings

Several themes were identified to answer our research questions regarding what aspects of WSAs existed previously within the regions; the extent to which the two WSAs constituted a new working approach; and methodologies used for implementation and their effectiveness. The first two themes related to what existed previously and the extent to which WSA were new, these included: WSA and existing ethos and practice; and broad perspectives on WSA/pilot approach. The remainder of the themes related to implementation and effectiveness, these themes included the process of implementing a WSA; impact of WSA; meeting community need/community and stakeholder involvement; barriers; and enablers. Each theme is described below.

### WSA and existing ethos and practice

When participants were first asked about what a WSA was, they identified that, for them, a WSA approach was about ‘changing beliefs’ of those engaged and working in the system, but there was little mention of the complexity of factors influencing healthy weight, or changing the focus from individualistic to wider systemic influences on behavioural patterns. One person identified that a WSA was ‘*Focused on inequality, and for looking at the overall approach of how we deliver it to people. So being quite person-centred, thinking outside the box, not being judgemental*’. (Location A Time point 2 Interview). It was evident that there were mixed views towards how the WSA fits with existing ethos, knowledge, and practice. This ranged from some participants having no previous knowledge of WSA working while others viewed the WSA as a familiar concept:

*whole systems in many ways isn’t necessarily a new concept or certainly isn’t in Scotland and many other areas, it’s been used in things like youth justice and stuff for a while, so I think there was a degree of awareness in terms of that kind of systems thinking about kind of complex issues* (Location A Time point 1 Focus Group).

On closer inspection, there was broad knowledge of what a WSA is; however, this was not backed up with a detailed description or understanding. Not being aware of what a WSA entailed led to confusion where one participant felt the need for action to target obesity instead of ‘structures’; and that the community had not bought into the WSA or the idea of systems change.

Despite some participants not being fully familiar with what a WSA entailed, it was felt that the approach fitted with existing ethos, goals, and ongoing work, and that there was value in applying WSAs given the scale of obesity and limited effectiveness of previous approaches. Ahead of further roll-out, it was reported that WSA obesity work may provide useful information ahead of scale-up to further areas of public services, and in some cases has prompted engagement with long-established complicated systems across government departments:

*because normally I don’t think we would do that [i.e., spend a large amount of time on planning and engagement with stakeholders], it’s like you kind of leave those systems alone because they’re too big, they’re too established, they’re too prescriptive almost. So, I think that’s been the difficulty for us kind of here is that we are trying to do that and that’s perhaps a bit alien to us even those of us who’ve worked, like been around forever* (Location B Time point 2 Focus Group).

It was apparent from the participant that there was a change in belief about how they can engage in the system and by adopting a WSA, a new way of working or method of engagement with the system was required. A further example:

*Yeah, for me I think this example is something perhaps like normally we would just let it go as something that’s kind of unachievable, but for me I suppose this is perhaps a test of the method to see like how do you do it?* (Location B Time point 2 Focus Group).

### Process of implementing a WSA

Despite the implementation process being in part reliant on stakeholder engagement, limited engagement was reported, alongside difficulty in involving relevant representatives from partner organisations. Some of the difficulty related to participation in group sessions with remote/virtual workshops being considered a challenge.

Variable types of communication to stakeholders generated an initial sense of the WSA having momentum, and then losing this, leading to uncertainty about the next steps and what contribution was required of stakeholders. Overall, there was a perception of slow progress throughout the WSA implementation process primarily due to COVID-19, but also impacted by leadership changes in both areas:

*I think we could probably have done more on just that sort of two-way communication with stakeholders over the longer term but it’s been quite hard because the progress has been, I mean I don’t know what we could have done differently because the progress has been slow* (Location B Time point 1 Focus Group).

### Experience of the Leeds Beckett Methodology (Location B only)

The structure provided by the LBM was found useful for implementation. The LBM was viewed as a new process and way of working, and many had no previous experience of implementing it. Some members who had received LBM implementation training had changed roles and were not involved throughout the full duration of the project, which meant those without training faced some barriers, mainly uncertainty in how to follow the process. Some viewed the implementation of the LBM as complex and some others as too abstract or theoretical in content. There were examples of when those in Location A rationalised needing to diverge from using the LBM to fit with their way of working and their existing system. However, in Location B, following each phase of the LBM was upheld and although it was perceived to be slow to implement, and at times a challenge to integrate or explain to the participants (e.g. Action Scales Model), it was considered a crucial part of the process.

Overall, views about the LBM were mixed, seen by some as challenging to work through, but by others as offering a useful step-by-step framework. While the LBM can be used as a structure for partnership working, its structure was perceived as complex, as the methodology needed subject expertise, and communicating this suitably to other colleagues or stakeholders during workshops was difficult. This was further impacted by changing communication medium:

‘*so it was the start of it that was quite tricky to get your head around it*’ and ‘*people can only stay in an online workshop for a limited amount of time so you just end up with like disorganised thought*’ (Location B Time point 1 Focus Group).

Some would have preferred more instruction on the LBM.

The LBM includes two workshops. A common view was that the time elapsing between these was too long, reducing momentum following the first, contributing to attrition, with fewer people returning to the second. It was felt that the lack of activity between workshops inhibited progress and that time between subsequent future workshops should be reduced.

### Impact of WSA

#### Impact on partnership and collaboration

WSA implementation was viewed as promoting understanding of why collaboration and partnership across the system was important to improve health and wellbeing of the local community. Bringing together the CWG and other stakeholders supported establishment of new networks, engagement opportunities and knowledge about communities for whom outcomes are intended to be improved: ‘*there’s definitely been connections made I would say just from the work that we’re doing that probably weren’t there, or they might have been there but maybe people just haven’t really nurtured them*’ (Location A Time point 2 Focus Group). The new networks were also viewed as strengthening partnerships and opportunities for collaboration, post the COVID-19 pandemic.

#### Understanding of the complexity of taking a WSA and acknowledging challenges

Driven by limitations in understanding a ‘working’ WSA, it was apparent that some members may be insufficiently focused on systems, and rather focusing on event-level activities. Event-level activities refer to reactive actions that offer little leverage for system change, often thought of as quick fixes. Examples include delivery of interventions, campaigns, or awareness raising to the public (e.g. educating people about high-sugar beverages, provision of weight management programmes, implementation of physical activity in schools). It was acknowledged that WSA work was challenging and with progress still to be made, necessitating a long-term approach and perspective. There was also recognition that the steering groups were at the early stages, resulting in limited direct impacts on obesity, but rather working together to establish a system for future impact. In this quote, the long-term impact of developing the system is acknowledged:

*it has kind of made you, is there this whole systems approach is there a way that we can identify it or use that methodology in other areas of our work, maybe it is an opportunity for us to build up other partnerships with like, on different topics or different subjects, try and, and like [person’s name] said, raising a kind of small amount of awareness with the key stakeholders that’s been involved so far that there is something happening* (Location B Time point 1 Focus Group 1).

#### Meeting community need/community and stakeholder involvement

Participants were not convinced their WSA met community need due to insufficient involvement of people from communities; some wanted increased involvement of community members, but COVID-19 had restricted this:

‘*so doing a sort of other workshop with just, you know, community members being able to say to them well this is something that we’re hoping to do within your area, these are some actions that we’re hoping to develop, is it something that you think’s good or do you think there’s enough of something or would you change something about it. I would’ve liked that to happen*’ (Location A Time point 2 Focus Group). There was some evidence of anxiety about public involvement, as there would be no-event level interventions in the near future. There was some concern that consultation without immediate intervention or action would not be welcomed by communities.

Some groups, which may have benefitted the WSA, were identified as missing; for example, health visitors, midwives, and the education sector in Location A; social work and housing in Location B. It was also felt that younger people could have been better represented: ‘*I think the people that you’re trying to target and the people you’re trying to improve the lifestyle choices of, those people weren’t at that meeting*’ (Location B Time point 1 Interview). Positively, the WSA was viewed as an opportunity to identify wider and important stakeholders, and consider how to subsequently engage them.

### Enablers

Identified enablers and barriers to implementing a WSA to diet and healthy weight are summarised in Table 1.

**Table 1:** Summary of enablers and barriers to WSA implementation

Enablers	Barriers
• Personal interest in WSA	• Covid impacts
• Links and relationships with key person/people	• Previous experience of consultation without action;
• Belief in approach/perception that WSA might lead to ‘real’ change	• Limited funding/constraints on use of funding
• Higher/strategic/national-level drive, change, and buy-in	• Daunting nature of workshops
• Sustained impetus in the WSA process	• Staff turnover
• Funding availability (appropriate and adequate)	• Tendency to revert to old ways of working
• ‘Real’, tangible action to encourage engagement	• Difficulty engaging community/stakeholders
• Engaging the ‘right’ people	• Lack of local leadership
• CWG comprising individuals with diverse expertise	• ‘Taboo’ nature of diet and healthy weight as a topic in community
• Communication and messaging of WSA work (e.g., in accessible/understandable language/terms)	• Publicity, marketing, framing of WSA• Multiple competing messages about diet and healthy weight
• Existing governance structures to build on;	
• Community buy-in	

WSA: whole systems approach; CWG: core working group;

#### Enablers of a WSA

There were many enablers for adopting a WSA in both LAs. Utilising existing structures where a local governance group had already been set-up, was viewed positively.

There were previous community action plans that were built on to form a new action plan.

*we already knew what sort of things were actually important to them in terms of broader health and wellbeing, so wasn’t then an opportunity for us to really build on some of that, so that kind of formed the basis if you like for our sort of informed future action plan* (Location A Time point 1 Focus Group).

Participants described how their current role and involvement in Type 2 diabetes prevention work provided good context for being involved in the WSA, and that was also good alignment with their organisation’s aims and objectives.

*So I want to obviously try and do something like that. So, as I said, it fits in well with all of our school, it fits in well with what I was interested in and it fits in really well with my subject* (Time point 1 Location B interview).

The WSA was also perceived to fit within pre-existing work on obesity where a system was already established – for example, coordination networks between services, holistic approaches. Involvement was also viewed positively due to funding availability for the establishment of the WSA, a focus on Scotland’s WSA pilot areas by Public Health Scotland, and opportunity to impact beyond diabetes prevention: ‘*funding is very, very important because it wouldn’t get done if there was no funding, people would not come, organisations would not come onboard if there was no funding*’ (Location A Time point 1 Focus Group)

The benefits of funding were also mentioned at time point 2 data collection – ‘*funding was the gift, you know, funding’s what got people to where we are*’ (Location A Time point 2 Focus Group).

*We’re talking about Type 2 diabetes here, but you could use, you could use coronary heart disease or stroke where the things that we’re trying to tackle through the whole system approach will have an impact beyond Type 2 diabetes, so that was something that really drew me to it* (Location A Time point 1 Focus Group).

Some participants expressed personal interest in WSAs and were excited to get involved locally to create a ‘real’ difference and improve people’s lives:

*I’ve got a personal interest in nutrition so I suppose I was coming at it from that angle and I think the sort of social determinants of diet and weight are really interesting, so as soon as we started looking at where we were going to, where we were going to think about hosting the pilot you know, sort of really did drive home that link between deprivation and determinants of you know, risks, risks for diabetes in the future.* (Location B Time point 1 Focus Group).

Pre-existing positive relationships with people already involved in the WSA contributed to some accepting invites to join the WSA initiative. It was also viewed that buy-in from strategic level partners contributed to real change and solutions to systemic barriers encountered previously:

*the difference with this was that there is that buy-in from that it’s strategic level and for me in particular even back in like pre, when public finances were good we still struggled to get involvement from like Social Work and NHS colleagues at that time, so I know it’s not a clinical project but I felt that would be one kind of positive from this that we’d be able to pull those services in the mix and again just try to look at the bigger picture stuff and then see where it goes* (Location B Time point 1 Focus Group).

Benefits accrued where the area WSA lead was familiar with the local community and its needs; pre-existing connections and relationships of the lead also positively impacted initial WSA adoption. The importance of the lead and their seniority was a motivator for WSA involvement. The regional Public Health Programme Director started the process, and this was seen as particularly encouraging for steering group members as it led to the community working with statutory organisations.

There was a perception that adequate resource and training would be provided and the establishment of formal procedures reassured participants of quality assurance in the process: ‘*we were asked to kind of go through rightly a kind of governance and accountability process around putting together an application*’ (Location A Time point 1 Focus Group).

‘*we had you know, two and half days altogether of training on the methodology and the principles and how to hold the workshop so I had that advantage*’ (Location B Time point 1 Focus Group 1).

Connections and relationships between steering group members and other stakeholders were viewed as important.

‘*it’s knowing the agencies wider than (community name mentioned) that potentially could contribute to those conversations, and I think that’s, that’s been a strength too*’. (Location B Time point 2 Focus Group). Furthermore, the composition of the workshops was quite varied, and this aided communication and the establishment of potential partner organisations. It was recommended that the WSA CWG needed to have as diverse a membership as possible.

#### Barriers to a WSA

Barriers to adopting a WSA included some stakeholders being unfamiliar with a WSA and having consequent feelings of apprehension about engaging. Further elaboration indicated that use of nuanced WSA language was perceived as off-putting. For example, the language used to describe WSA mapping was perceived to need to be simplified and explained more clearly for the policy makers and the public. Caution was advised around the workshops as some individuals were more vocal at them, and sometimes more forceful with pushing their ideas forward for implementation.

While exacerbated by COVID-19, digital exclusion needs to be addressed to allow engagement with both key stakeholders and the wider community. Digital delivery was viewed as a challenge by a facilitator given the inherent visual aspect of mapping at the workshops:

*it’s a really difficult method to do online, to do on Zoom when people are still getting the hang of Zoom and it’s probably the worst method to do online because of the visual aspect of it and how sort of wide the mark goes.* (Location B Time point 1 Focus Group 1).

Changing roles and staff turnover was viewed with concern, particularly where WSA leads changed, as happened in both locations; this contributed to a loss of momentum. It was recognised that this was attributable to COVID-19 and the demands on public health teams at that time, with many staff being ‘stretched’. These factors also negatively impacted on community engagement.

There was a view that within communities there was limited awareness about WSAs. It was felt that the public did not perceive obesity as a system issue but rather one driven by individual decision-making; this may be improved by community education around WSAs and weight stigma. In addition, some participating professionals did not share a common understanding of inequality and poverty, and so making decisions about public involvement was queried: ‘*that core working group absolutely it was not right that that was made up of like public service workers because actually some of the attitudes and the understandings about inequality and poverty were way off the mark*’. (Location A Time point 1 Focus Group)

The time elapsing between the two workshops was identified as a barrier, given its impact of reducing momentum in establishing and maintaining stakeholder engagement:

*so I think once we got into it we kind of got a bit of an explanation but I think it was just missing that next step, I guess for me I expected there to be a follow-up pretty quickly to that, probably around September time, October time, like it’s kind of been left, if that’s the best way of putting it, it’s kind of been we’ve done this, I don’t know if [name of participant] agrees, we done this in the summer and it’s like well we’ll follow-up with and then nothing really pretty much.* (Location B Time point 1 Focus Group 2)

Others, reflecting at time point 2, identified it as important to establish where stakeholder agendas compete, and how to resolve such to ensure ‘buy in’:

*I think if I’m honest the biggest is just people’s priorities, if it’s not something that they don’t see a direct impact or effect on their service, on their topic of work, they won’t want to come on board with it, and it’s quite difficult to get everyone on the same page because although everyone might be round that table everyone does come with their own agenda, so I think that, yeah, for me on paper it sounds amazing, in practice you still have some of that silo working and it’s really hard to try and bring that all together.* (Location A Time point 2 Focus Group).

There was often pessimism around public messaging effectiveness, given the saturation of health messages: there was concern about whether a WSA would ‘fit’ and its acceptability. Given that the WSA was introduced at the time of COVID-19, this may explain the view that there were many health messages already in the public domain.

### COVID-19 pandemic and the effect on WSA Steering Group progress

The COVID-19 pandemic had considerable impact on engagement with whole systems working. This was reflected in staff being re-deployed to support efforts focused on addressing the pandemic. Consequently, the impetus for WSA progression was downgraded. COVID-19 also restricted how the steering group could engage partners and community representatives with the constraint to move online restricting involvement. Referring to the delivery of the workshops, participants said ‘*it had to be done virtually because of all the Covid restrictions . . . I think if Covid hadn’t had, had been there at that time it would have been a really different event*’. (Location A Time point 1 Focus Group).

### Longitudinal views of WSA

Participants recognised that after setting up the CWG and facilitating the workshops, that the scale of implementing the WSA to target healthy weight and diet is substantial and that uncertainty surrounding funding can negate progress. There was a shared view that funders want to see change in a shorter period of time, yet WSAs are long term, and that some structures in a system are not likely to change, such as the funding timelines and expectations of creating a change in health outcomes. It was reported by some that a lack of funding long term can put a burden on human resources within a system, as it needs to be completed yet there are few people who stay involved long term due to staff turn-over or community group leads focusing on other prioritised actions (e.g. applying for funding). There was a positive view that the ongoing harnessing and connecting of networks is beneficial for planning and finally implementation. The long-term continued engagement of senior management in public health was perceived as being required as higher-level funding decisions, and human resource allocation are out of the CWG’s control.

### Activity timings from monthly surveys

Total time of activity, and their indicative costs, by location are reported below in Table 2 and plotted in Figure S1 (in Supplementary materials). The percentage of total time spent on each activity type is reported below in Table 3 and plotted in Figure S2 (in Supplementary materials). Proportionately, most time is spent on events and email administration.

**Table 2:** Total time (hours) spent on each activity by location and indicative total cost (£ 2021/2022 values) of employing staff to deliver these activities.

Location	Meeting/ events (attending/ planning)	Email admin	Phone calls	Reading	Other activity	Total time	Total cost (£)
A	118	20	2	4	6	150	6320
B	49	13	0.03	10	5	77	3219

**Table 3:** Percentage of total time spent on each activity by location.

Location	Meeting/ events (attending/ planning)	Email admin	Phone calls	Reading	Other activity
A	78	13	2	3	4
B	64	17	0	13	7

## Discussion

We aimed to assess implementation of WSA to diet and healthy weight in two council areas of Scotland, exploring enablers and barriers over time. One area followed the LBM of implementation, while the other used a hybrid model incorporating existing working systems. Factors that supported the pilot sites to progress with their WSAs included: stakeholders’ belief in WSA effectiveness; positive relationships between key personnel; ‘buy-in’ by the public health authority at a national level; funding availability; CWG comprising individuals with diverse expertise; effective communication; supportive existing governance structures; and community buy-in. Several of these enablers have been identified previously.^6,9–12^ A unique contribution of this article was in the assessment of enablers at two time points: demonstrating their relevance between initial WSA set-up and attempted implementation up to 1 year later.

Several highlighted barriers would be important to consider for those aiming to adopt a WSA to diet and healthy weight. These include: appropriate funding; minimising staff turnover (or planning ways to mitigate its effects); and ensuring adequate training in WSA is available at all stages – not just its inception (otherwise knowledge/familiarity is lost when staff leave). What might be considered appropriate or sufficient funding to achieve success in taking a WSA to diet and healthy weight is not yet known, but as an example, the Amsterdam Healthy Weight programme,^21^ which has shown indicators of a significant impact on rates of obesity^22^ involved sustained investment of millions of Euros over many years.^23^

Training approaches need to address apprehension around perceived WSA complexity. A suitably trained and confident ‘workforce’ may help combat the identified tendency to revert to ‘old ways’ of working, noted as a risk at time point 2 in our study. Our findings also showed that it would be beneficial for those adopting a WSA to integrate time for WSA training for all involved staff. Systems-based approaches involve the adoption of a broad perspective that focuses on the collective effects of a wide range of factors – such as people’s beliefs, motivations, and capabilities; their social networks; societal structures and environmental exposures; therefore, the training offered to staff needs to take account of these factors and ensuring staff are aware of what a WSA entails and are negated from a reverting back to usual ways of working, that maybe intervention targeted.^12^ Training delivery also needs to be robust to staff changes and turn-over – for example, it cannot only be delivered at one time (i.e. usually at the beginning). Considering ongoing support or mentoring for taking a WSA may also help to combat issues to do with staff confidence, competence, and turnover. According to the recommendation from a recent report by the Academy of Medical Sciences on what’s next for WSAs in public health, there was an admittance that more work and evidence gathering is required, but importantly there is a need to develop a global community of practice for sharing what works in systems approaches in public health.^12^ One way of building staff confidence and capability will be through connecting policymakers and public health practitioners with researchers. Such a community could provide a platform to share evidence, support the use of new methodologies, and promote the use of existing approaches for the betterment of implementation and to facilitate change.

To our knowledge, this is the first independent longitudinal process evaluation of the LBM where participants have been followed up 1 year later. As the full components of the model were not implemented (phases 4–6 – see Figure 1), further follow-up is required to ascertain the complete value of the LBM; however, we conclude that for the initial phases of WSA set-up, the LBM was advantageous in Location B compared to not applying a clear guidance model as in Location A. It was also apparent that the model or approach used to implement the WSA should be aligned with the existing ethos of the organisations’ goals and targets. We also suggest an emphasis be placed on reminding staff and stakeholders about the long-term commitment required to successfully adopt a WSA where focus should be on system development, stakeholder engagement, system mapping and planning, and not event-level programme development.

As the WSA considered here were delivered during COVID-19, with its required move to remote working, it is important that lessons are learned from this experience. The importance of staff leadership was highlighted to promote collective working and the need for stakeholder workshops to be delivered face-to-face. From the interview and focus groups at time point 2, a novel finding is that use of language like ‘type II diabetes’ was a hurdle to engaging partners; the narrative and language used to engage partners requires careful consideration. Participants also suggested the use of marketing to inform the public about WSAs to diet and healthy weight. Such marketing may support engaging young people and end-user groups.

The analysis of activity timings reported on the monthly surveys showed that email communication and delivering events (in that order) were the most commonly reported activities used to progress WSA implementation. Costs of funding staff to deliver on such a broad intervention over such a duration seem relatively modest (£6.2K and £3.2K – see Table 2), though these figures exclude other costs (e.g. pilot funding) and are subject to under-reporting.

We sought to utilise EMA to provide a longitudinal and quantitative perspective to our evaluation. Unfortunately, responses rates were very low and so did not provide suitably robust data to be reported here. This fits with findings from our systematic review, which highlight the difficulties of evaluating the long-term impact of WSAs.^10^ However, we include details of the EMA in the Supplementary materials, so other researchers can build from this work. We believe a successful EMA would be fruitful because data can be collected more frequently, in close proximity to when an activity occurred in the system, and collected via survey so relatively low cost, with little intrusion on time. A successful utilisation of EMA would require overcoming the challenge of evaluating a diffuse intervention over long periods, engaging participants for whom it is likely not a main focus.

## Limitations

The study was conducted during the COVID-19 pandemic so plans at each location on implementation were consequently adjusted – this may have impacted the number of participants recruited to the WSA workshops, subsequently impacting recruitment to the evaluation interviews and focus groups. The study design was longitudinal; however, further time would be required to establish what impact the WSA can have on diet and healthy weight outcome behaviours. Responses to the monthly surveys (detailed in Supplementary Table S1) were few so the indicative costs should be interpreted with caution, as it is likely these are significant under costings (missing the activity delivered by staff not completing the survey and, here, excluding the activity of staff working across both areas). In Location A, a hybrid model was used that relied on the functions of the existing system. It has been argued that a WSA aim is to perturb an existing system^12^ not just ‘fit in’ to an already established system. However, as systems approach application lies upon a continuum from low, medium, and high, we were satisfied that the hybrid model in Location A was at least at a low level of application. It had the following characteristics: (1) identified the groups of people, institutions, and structures that influence diet and healthy weight in the area; (2) mapped the relationships of these ‘agents’ or ‘factors’ with target populations and with each other; and (3) carried out evaluations that capture multiple outcomes and process data (our current evaluation; Jebb et al.).^12^ Location B shared similar characteristics with Location A; however, they also used the LBM, and Action Scales Model to identify potential points of intervention, which may mitigate or enhance the impact of potential interventions. To conclude, this study provides the first independent longitudinal process evaluation of WSA that have included the LBM, and offers useful insights into how to maximise likely successes in the early phases of setting up and implementing a WSA to tackle diet and healthy weight.

## Supplemental Material

sj-pdf-1-rsh-10.1177_17579139231203858 – Supplemental material for Whole systems approach to diet and healthy weight: a longitudinal process evaluation in East Scotland Click here for additional data file. Supplemental material, sj-pdf-1-rsh-10.1177_17579139231203858 for Whole systems approach to diet and healthy weight: a longitudinal process evaluation in East Scotland by G Breslin, W Wills, C Bontoft, O Fakoya, H-A Greco, N Lloyd, AP Wagner, A Wellings, S Harding and KE Brown in Perspectives in Public Health
