# Supplementary material for: Whole systems approach to diet and healthy weight: a longitudinal process evaluation in East Scotland
Source: Perspect Public Health. 2023 Oct 30;143(6):347–57. doi: 10.1177/17579139231203858 (PMC10683340; doi:10.1177/17579139231203858)
Supplement: sj-pdf-1-rsh-10.1177_17579139231203858 – Supplemental material for Whole systems approach to diet and healthy weight: a longitudinal process evaluation in East Scotland [file sj-pdf-1-rsh-10.1177_17579139231203858.pdf]

Supplementary materials

EMA detail

EMA is a structured self-report technique used to assess attitudes, behaviours, and situational factors as they occur. Typical EMA studies require participants to complete short self-report questionnaires to capture in-situ experience. Our EMA consisted of monthly surveys from December 2021 to December 2022, and included ten questions assessing levels of agreement with a series of statements scored either on a Likert scale [coded one ('strongly disagree') to five ('strongly agree')] or scale of agreement (recording a value of 0-100, with 100 corresponding to maximal agreement). See Table S2 for details of the questions and their response scale. We believe this to be the first time EMA has been applied to assess WSAs to diet and healthy weight. Since response rates (see Table S1 were very low, particularly in later months, we do not report the associated information further.

Table S1: Number of surveys completed, separated by authority (including those that working across authorities). This only includes people who clicked the survey link AND responded to questions ('engaged' participants).

Date	Location A	Location B	Location A + B	Total
Dec-21	1	8	1	10
Jan-22	0	7	1	8
Feb-22	0	6	2	8
Mar-22	1	4	1	6
Apr-22	0	4	1	5
May-22	3	3	1	7
Jun-22	1	4	1	6
Jul-22	3	2	1	6
Aug-22	2	3	0	5
Sep-22	2	1	0	3
Oct-22	2	0	0	2
Nov-22	2	2	0	4
Dec-22	2	0	0	2
Jan-23	2	0	0	2
Mean (engaged) monthly responses	1.50	3.14	0.64	5.29

Table S2 – Questions asked in monthly surveys and associated response scale, constituting the EMA (Q1-12) and the Health Economics (Q13).

	Question	Response scale
1	In the last month, how involved have you been in WSA work in your local area?	0- They have not been engaged at all<br>50- They have been engaged a little<br>100- They have been very engaged
2	In the last month, in your view how well has the Whole Systems Approach (WSA) functioned in your local area?	0- They have not been engaged at all<br>50- They have been engaged a little<br>100- They have been very engaged
3	In the last month, how engaged do you feel the majority of WSA stakeholders have been with the WSA in your local area?	0- They have not been engaged at all<br>50- They have been engaged a little<br>100- They have been very engaged
4	In the last month I have felt that I am a valuable member of the WSA team in my local area	0- Strongly disagree<br>50- Neither agree nor disagree<br>100- Strongly agree
5	In the last month I have felt supported by my organisation in my work on the WSA	0- Strongly disagree<br>50- Neither agree nor disagree<br>100- Strongly agree
6	In the last month I have felt supported by other stakeholders in my work on the WSA	0- Strongly disagree<br>50- Neither agree nor disagree<br>100- Strongly agree
7	In the last month My work has been core to the development of a WSA	0- Strongly disagree<br>50- Neither agree nor disagree<br>100- Strongly agree
8	In the last month I have been optimistic about the benefits of adopting a WSA to the local area	0- Strongly disagree<br>50- Neither agree nor disagree<br>100- Strongly agree
9	In the last month I have felt engaged in developing the WSA	0- Strongly disagree<br>50- Neither agree nor disagree<br>100- Strongly agree
10	In the last month I have felt competent that I can contribute to the delivery of the WSA in the local area	0- Strongly disagree<br>50- Neither agree nor disagree<br>100- Strongly agree
11	In the last month, please describe in **ONE** word your overall experience of working within the WSA:	<Free text>
12	In the last month, what planning, coordination, delivery or implementation activities have occurred as part of the local WSA partnership?	<Free text>
13	In the last month, how much time have you spent on activities aimed at progressing the WSA?<br>- Meetings / events (attending or planning)<br>- Email administration<br>- Phone calls<br>- Reading documents	Estimated time spent on this type of activity over the month (minutes):<br><br><br><Free text><br><Free text><br><Free text>

	- Other – please detail: ….....................................	<Free text><br><Free text>
14	Please use the space below to outline anything else you would like to tell us about your work with the WSA in the last month.	<Free text>

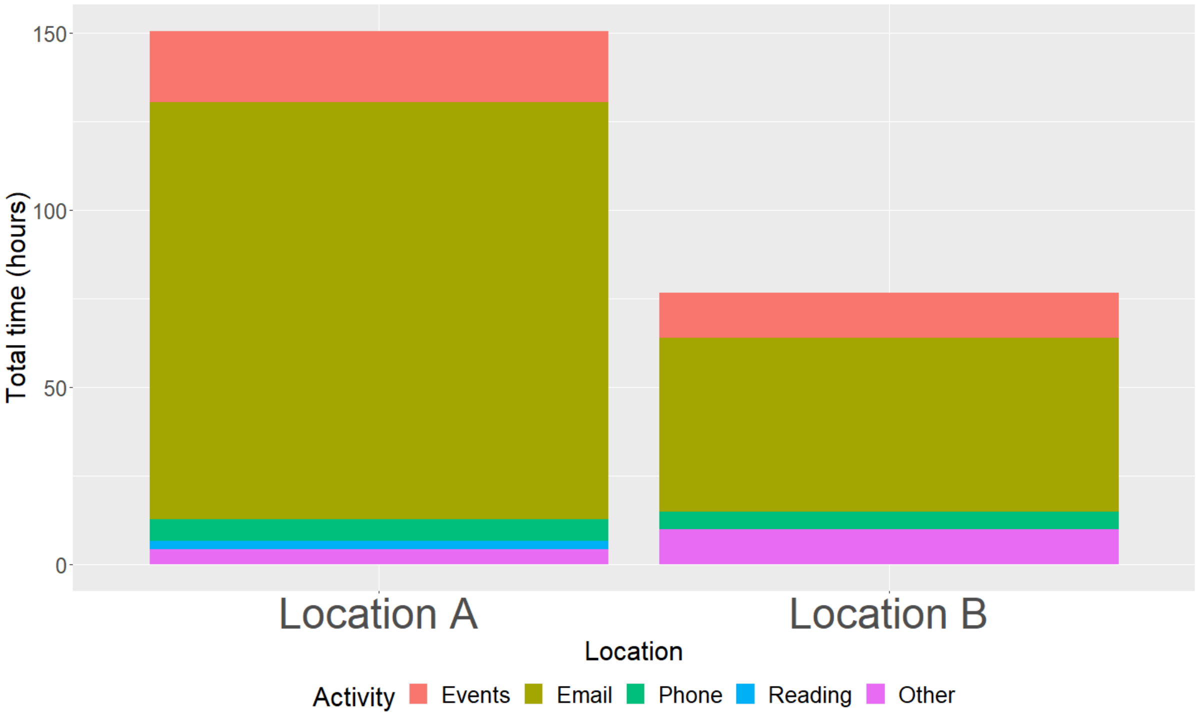

Figure S1: Totals of time (hours) spent on each activity by location.

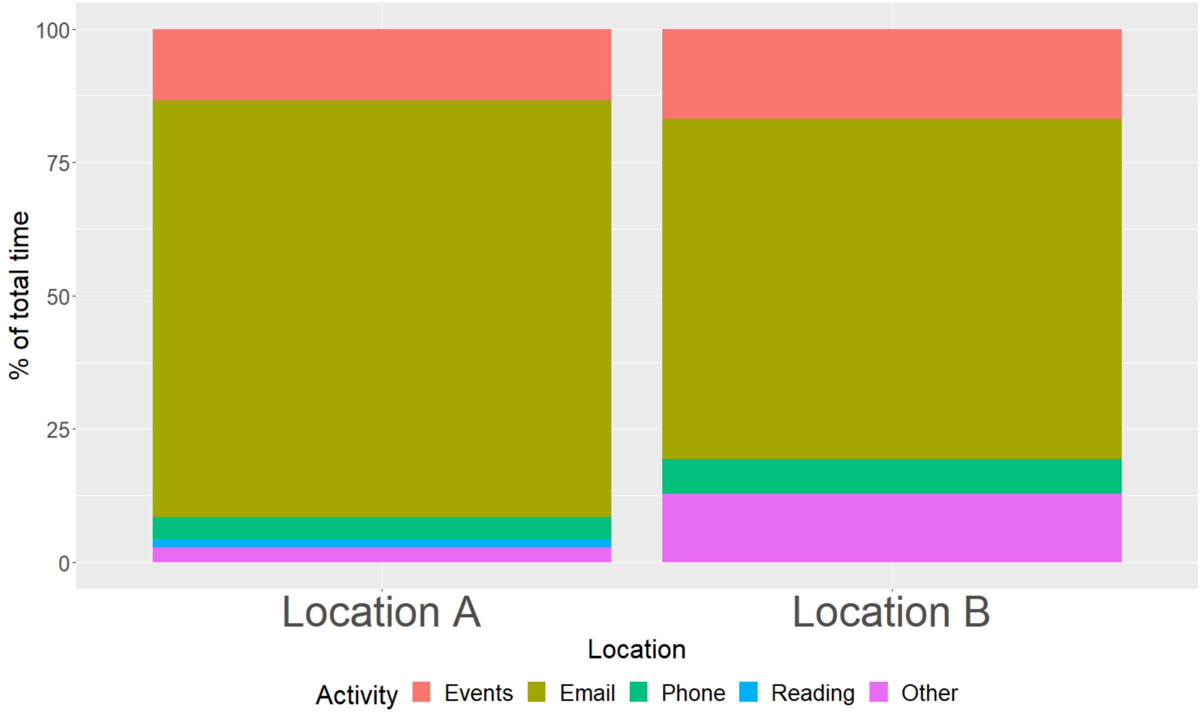

Figure S2: Percentage of total time sent on each activity type for each location.
